# Post‐COVID‐19 Public Health Challenges: Insights Into Post‐COVID Symptoms, Stigma, and Post‐Pandemic Recovery Support From the Vietnam Experience

**DOI:** 10.1002/puh2.70396

**Published:** 2026-10-04

**Authors:** Binh‐Minh Le Thi Nguyen, Truc Thanh Thai, Tri Nugraha Susilawati, Thanh Chi Vo, Mai Quynh Pham, Anh‐Thu Trang Thai, Nguyen Thao Dang, Nhan My Le, Thuy Anh Bui, Minh Cuong Duong

**Affiliations:** ^1^ University of Medicine and Pharmacy at Ho Chi Minh City Ho Chi Minh City Vietnam; ^2^ Nguyen Trai Hospital Ho Chi Minh City Vietnam; ^3^ Faculty of Medicine Universitas Sebelas Maret Surakarta Indonesia; ^4^ School of Population Health UNSW Sydney Sydney New South Wales Australia

**Keywords:** post‐COVID‐19, post‐COVID symptoms, quality of life, social stigma

## Abstract

**Objectives:**

This study aimed to assess the burden of post‐COVID‐19 symptoms, social stigma, and quality of life, and to examine their interactions among individuals recovering from COVID‐19 in Vietnam.

**Methods:**

A cross‐sectional survey was conducted from March to June 2022 among individuals who had recovered from COVID‐19 within 1–3 months prior. Participants completed a self‐administered questionnaire capturing demographic and baseline characteristics, health status during infection, post‐COVID symptoms, perceived social stigma using the five‐item COVID‐19 Perceived Discrimination Scale (C‐19‐PDS), and quality of life using the EQ‐5D‐5L. Multivariable logistic regression was used to investigate the relationship between social stigma and perceived decline in quality of life.

**Results:**

Among 764 participants, 55.4% were female, and 45.4% were under 30 years old. Most participants (81.3%) reported at least one of 27 post‐COVID‐19 symptoms, 27.5% experienced social stigma, and 38% reported a decline in quality of life. Perceived social stigma was significantly associated with reduced quality of life (adjusted OR = 1.81; 95% CI 1.20–2.73).

**Conclusions:**

Post‐COVID symptoms and social stigma are common among COVID‐19 survivors, and social stigma significantly impacts post‐recovery quality of life. Comprehensive support from families, communities, and authorities is essential to promote full recovery and social integration.

## Graphical abstract

Post‐COVID‐19 symptoms and social stigma were common among COVID‐19 survivors in Vietnam. Notably, perceived stigma is significantly associated with poorer quality of life. These findings highlight the need for comprehensive post‐pandemic recovery support to promote well‐being and social integration.

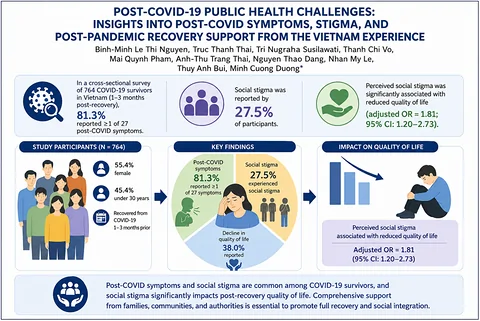

## 1 Introduction

Chronic infectious diseases impose a dual burden of persistent physical morbidity and social adversity. Visible symptoms and persistent misconceptions regarding infectivity often precipitate labeling, social avoidance, and discrimination [1]. This intersection of biomedical pathology and psychosocial exclusion severely compromises social reintegration [2].

Similar patterns have been observed during the COVID‐19 pandemic, which has globally affected over 770 million individuals, resulting in more than 7 million deaths since 2019 [3]. While most cases achieve clinical recovery, a substantial proportion of survivors continue to experience persistent physical and psychological impairments, indicating incomplete recovery [4]. These post‐COVID‐19 symptoms are associated with significant declines in quality of life, with up to 59% of recovered individuals reporting ongoing physical or mental health challenges [4, 5, 6].

Post‐COVID symptoms can manifest as fatigue, shortness of breath, joint pain, headache, and cognitive impairments [7]. Psychological consequences, including anxiety, depression, post‐traumatic stress disorder, and sleep disturbances, are also common [4, 5]. Older age and greater severity of the acute infection are consistently linked to poorer post‐recovery outcomes, likely due to reduced physiological resilience and preexisting comorbidities [4, 5].

Beyond health challenges, COVID‐19 survivors often face social stigma arising from fear, anxiety, and misunderstanding about the virus and post‐COVID consequences [8]. Stigma can lead to exclusion, discrimination, and difficulties reintegrating into society, further undermining mental health and recovery [8, 9]. Studies from developing countries have reported that 50%–70% of COVID‐19 survivors experience social stigma, which is strongly associated with lower quality of life [10, 11]. Despite this evidence, the relationship between COVID‐19‐related stigma and post‐recovery quality of life remains insufficiently explored.

In Vietnam, approximately 12 million confirmed COVID‐19 cases have been reported, with around 11 million recoveries [12]. However, data on post‐COVID‐19 quality of life from these patients and its link to social stigma are limited. To support post‐pandemic health monitoring and future pandemic preparedness, this study aimed to examine post‐COVID‐19 symptoms among recovered patients in Ho Chi Minh City and investigate the prevalence of social stigma and its association with perceived decline in quality of life.

## 2 Materials and Methods

### 2.1 Study Design, Context, and Participants

A cross‐sectional study was conducted from March to June 2022 among individuals who had recovered from COVID‐19 from 1 to 3 months prior. This period coincided with Vietnam's transition into the *new normal* phase, approximately 5 months after the country shifted toward adaptive living following the fourth pandemic wave [13]. Four districts of Ho Chi Minh City (Districts 5, 6, 8, and Hoc Mon) were selected using simple random sampling method. A multi‐stage sampling strategy was employed, ultimately incorporating 12 neighborhoods selected across 4 urban and suburban districts in Ho Chi Minh City, Vietnam. These districts were part of the COVID‐19 Levels 1 and 2 risk zones identified during the fourth wave and were chosen for their high population density, ensuring adequate demographic representativeness of the study population [14, 15].

The inclusion criteria included individuals aged 18–65 years, confirmed COVID‐19 diagnosis by primary healthcare centers, negative RT‐PCR or rapid antigen test result for SARS‐CoV‐2 within 1–3 months prior to the study, and provision of informed consent. Exclusion criteria included self‐reported neuropsychiatric conditions, communication limitations (e.g., hearing, visual, or language impairments), SARS‐CoV‐2 reinfection at the time of the study, or acute health conditions requiring hospitalization.

### 2.2 Study Procedure

Participants were recruited via multi‐stage cluster sampling, involving the random selection of 12 neighborhoods. A total of 1018 eligible individuals provided written informed consent before completing a structured, self‐administered questionnaire in Vietnamese.

### 2.3 Measurements

A self‐administered questionnaire comprised five sections: demographic and baseline health characteristics, health status during the COVID‐19 infection, post‐COVID‐19 health status, perceived social stigma, and perceived quality of life.

Sociodemographic characteristics included age groups (18–30, 31–40, 41–50, 51–60, and 61–65), gender, ethnicity (Kinh or others), education level (<high school, high school, and >high school), and marital status (single, married, or widowed/separated/divorced). Socioeconomic status was classified into three categories—lower, middle, and upper—based on household income thresholds relative to the media, reflecting the capacity for basic need fulfillment and discretionary spending.

The baseline health characteristics section included the number of COVID‐19 vaccination doses received (1, 2, or 3), the presence of chronic comorbidities (0, 1, or ≥2). The health status during the COVID‐19 infection section included place of treatment (home/hospital), severity of symptoms (mild or moderate/severe), and the duration of treatment (≤7, 8–14, and >14 days).

The post‐COVID‐19 health status section included recovery time (≤4, 4–8, and 8–12 weeks) and post‐COVID‐19 symptoms. The latter was referenced according to the NICE guidelines for the management of long‐term effects of COVID‐19 [16]. For the purposes of this study, the total 27 symptoms listed by NICE were classified into 8 groups: general symptoms (yes/no), which included body aches, anxiety, fatigue, hair loss, fever, and depression; neurological symptoms (yes/no), such as tingling/numbness in limbs, dizziness, headaches, forgetfulness, lack of concentration, and sleep disturbances; ear, nose, and throat symptoms (yes/no), including ear pain, sore throat, loss of smell, loss of taste, and tinnitus; respiratory symptoms (yes/no), which involve cough and shortness of breath; cardiovascular symptoms (yes/no), such as chest pain, palpitations, and arrhythmia; gastrointestinal symptoms (yes/no), including nausea, loss of appetite, abdominal pain, and diarrhea; musculoskeletal symptoms (yes/no), such as joint pain and muscle pain; and dermatological symptoms (yes/no), including rash and dermatitis.

Perceived social stigma was evaluated using the five‐item COVID‐19 Perceived Discrimination Scale (C‐19‐PDS) [17], which assesses survivors’ experiences of stigma‐related attitudes and discriminatory behaviors. Responses are scored on a 4‐point Likert scale (ranging from 0 = “never” to 3 = “always”), where higher cumulative scores indicate a greater intensity of perceived societal stigma.

The perceived quality of life was assessed using the EQ‐5D‐5L scale, which has been standardized for the general population of Vietnam [18]. This scale includes 25 subjective assessments across five dimensions: mobility, self‐care, usual activities, pain or discomfort, and anxiety or depression (five items per domain). Each item is rated on a 5‐point Likert scale, with higher scores reflecting greater impairment in daily functioning and, consequently, a poorer health‐related quality of life. For this study, any participant reporting impairment in one or more of these dimensions was categorized as having an impaired quality of life.

### 2.4 Data analysis

Data was analyzed using Stata version 17. Categorical variables are expressed as frequencies and percentages, whereas continuous variables are reported as means (standard deviation, SD) or medians (interquartile range, IQR), depending on their distribution. Univariable logistic regression was initially performed to examine associations between sociodemographic/baseline characteristics, clinical status, and post‐COVID‐19 outcomes (social stigma and quality of life). Variables demonstrating statistical significance (*p* < 0.05) in univariable analysis were subsequently included in multivariable logistic regression models to identify independent predictors and explore the association between social stigma and quality of life decline. Significance was set at *p* < 0.05.

## 3 Results

### 3.1 Baseline Characteristics of Study Participants and Their Associations With Perceived Quality of Life and Social Stigma

Figure 1 presents participant recruitment into this study. Of the 1018 eligible individuals, 810 completed the survey (79.6% recruitment rate). After excluding 46 incomplete responses, the final analytical sample comprised 764 participants. The cohort was predominantly female (55.4%), of Kinh ethnicity (89.0%), and aged ≤30 years (45.4%). Most participants had attained at least a high school education (79.8%), married (50.4%), and identified with the middle socioeconomic group (70.6%). Post‐recovery, 27.5% reported perceived social stigma, and 38.0% experienced a decline in quality of life (Figure 2). Multivariable analysis indicated that women (OR = 2.11, 95% CI: 1.56–2.86) and participants aged >60 years (OR = 2.52, 95% CI: 1.28–4.95) faced a significantly higher risk of decline in quality of life. Additionally, social stigma was significantly associated with age, marital status, and socioeconomic status (all *p* < 0.05).

**FIGURE 1:**
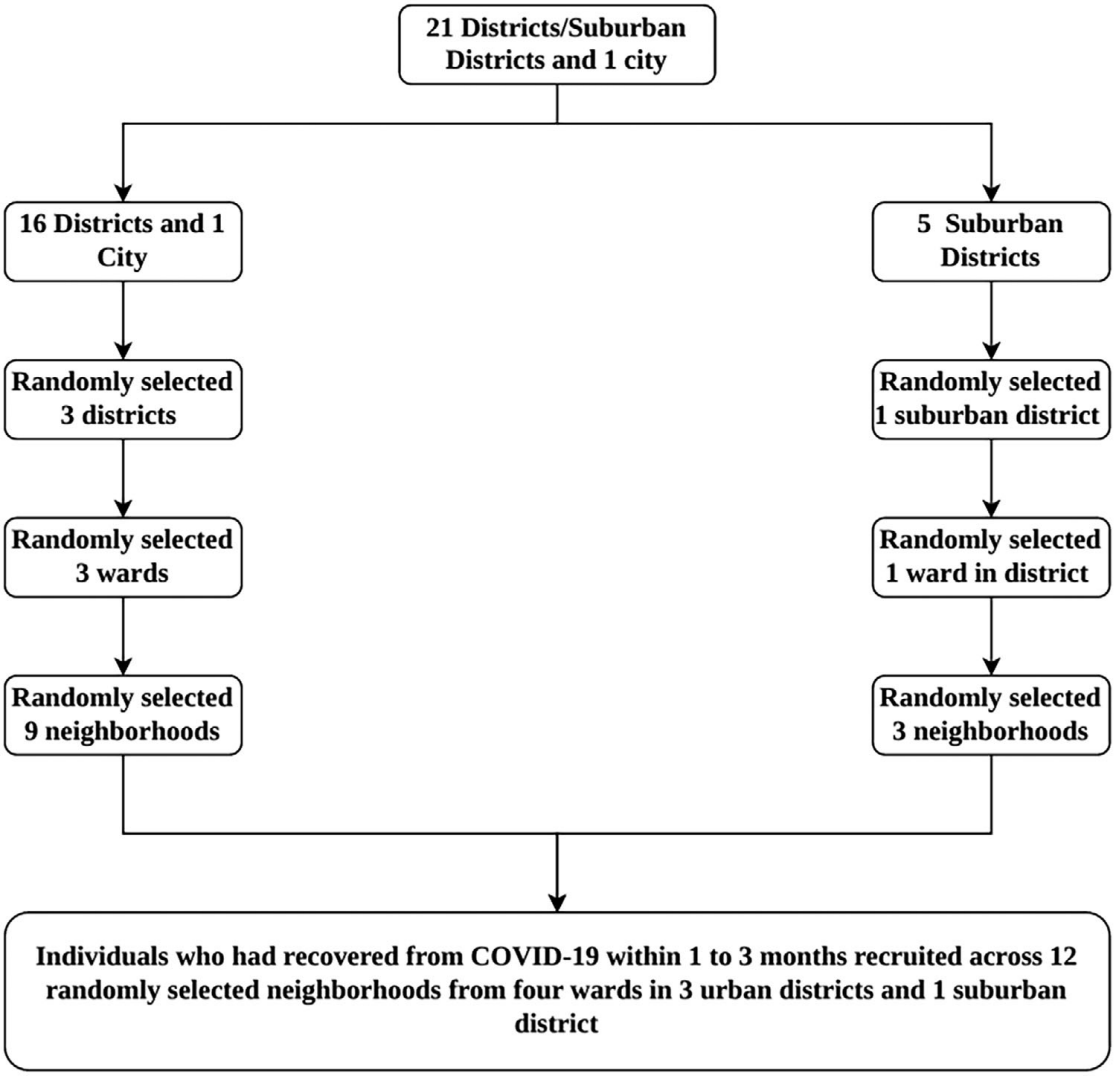
Flow chart of the participant selection process.

**FIGURE 2:**
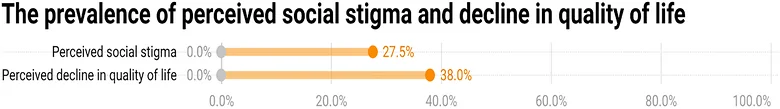
Proportions of perceived social stigma and decline in quality of life among 764 study participants recovering from COVID‐19 infection.

### 3.2 Clinical Characteristics and COVID‐19—Related Factors

As shown in Table 1, nearly all participants had received at least two vaccine doses (97.2%), with 49.8% having received three. Most reported no prior comorbidities (81.7%), experienced mild acute symptoms (92.9%), and were managed at home (96.3%). However, 48.2% reported a prolonged recovery period of 8–12 weeks.

**TABLE 1:** Demographic and baseline health characteristics of 764 study participants recovering from COVID‐19 infection, stratified by perceived decline in quality of life and social stigma (*N* = 764).

		**Perceived decline in quality of life**			**Perceived social stigma**		
Factor	Total *N* = 764 (%)	Decline *n* = 290 (38%)	*p* ^a^ value	OR^a^ (95% CI)	Yes *n* = 210 (27.5%)	*p* ^a^ value	OR^a^ (95% CI)
**Sex**							
Male Female	341 (44.6) 423 (55.4)	97 (28.5) 193 (45.6)	**<0.001**	1 2.11 (1.56–2.86)	98 (28.7) 112 (26.5)	0.486	1 0.89 (0.65–1.23)
**Age (years)**							
18–30 31–40 41–50 51–60 61–65	347 (45.4) 180 (23.6) 103 (13.5) 95 (12.4) 39 (5.1)	126 (36.3) 63 (35.0) 36 (35.0) 42 (44.2) 23 (59.0)	— 0.766 0.801 0.161 **0.007**	1 0.94 (0.65–1.38) 0.94 (0.59–1.49) 1.39 (0.88–2.20) 2.52 (1.28–4.95)	78 (22.5) 69 (38.3) 28 (27.2) 25 (26.3) 10 (25.6)	— **<0.001** 0.324 0.434 0.656	1 2.14 (1.45–3.17) 1.29 (0.78–2.13) 1.23 (0.73–2.08) 1.19 (0.56–2.55)
**Ethnicity**							
The Kinh Others	680 (89.0) 84 (11.0)	252 (37.0) 38 (45.2)	— 0.146	1 1.40 (0.89–2.21)	184 (27.1) 26 (30.9)	— 0.451	1 1.21 (0.74–1.98)
**Education level**							
H<High school High school >High school	154 (20.2) 205 (26.8) 405 (53.0)	66 (42.9) 85 (41.5) 139 (34.3)	— 0.791 0.062	1 0.94 (0.62–1.44) 0.70 (0.48–1.02)	40 (26.0) 49 (23.9) 121 (29.9)	— 0.653 0.363	1 0.90 (0.55–1.45) 1.21 (0.80–1.84)
**Marital status**							
Single Married Widowed/Separated/Divorced	352 (46.1) 385 (50.4) 27 (3.5)	122 (34.7) 157 (40.8) 11 (40.7)	— 0.087 0.524	1 1.30 (0.96–1.75) 1.30 (0.58–2.88)	83 (23.6) 119 (30.9) 8 (29.6)	— **0.026** 0.480	1 1.45 (1.04–2.01) 1.36 (0.58–3.23)
**Socioeconomic status**							
Lower Middle Upper	150 (19.6) 539 (70.6) 75 (9.8)	62 (41.3) 205 (38.0) 23 (30.7)	— 0.463 0.121	1 0.87 (0.60–1.26) 0.63 (0.35–1.13)	29 (19.3) 161 (29.9) 20 (26.7)	— **0.011** 0.211	1 1.78 (1.14–2.77) 1.52 (0.79–2.91)
**Number of shots of COVID‐19 vaccine given before infection**							
1 2 3	21 (2.8) 362 (47.4) 381 (49.8)	13 (61.9) 149 (41.2) 128 (33.6)	— 0.068 **0.012**	1 0.43 (0.17–1.06) 0.31 (0.13–0.77)	13 (61.9) 102 (28.2) 95 (24.9)	— **0.002** **0.001**	1 0.24 (0.10–0.60) 0.20 (0.08–0.51)
**Number of chronic comorbidities**							
0 1 ≥2	624 (81.7) 119 (15.6) 21 (2.7)	223 (35.7) 57 (47.9) 10 (38.0)	— **0.013** 0.269	1 1.65 (1.11–2.45) 1.63 (0.68–3.91)	167 (26.8) 39 (32.8) 4 (19.0)	— 0.180 0.434	1 1.33 (0.87–2.03) 0.64 (0.21–1.94)
**Place of treatment when infected with COVID‐19**							
Home Hospital	736 (96.3) 28 (3.7)	274 (37.2) 16 (57.1)	— **0.037**	1 2.25 (1.05–4.82)	200 (27.2) 10 (35.7)	— 0.323	1 1.49 (0.68–3.28)
**Severity of symptoms with COVID‐19**							
Mild Moderate or severe	710 (92.9) 54 (7.1)	258 (36.3) 32 (59.3)	**0.001**	1 2.55 (1.45–4.48)	179 (25.2) 31 (57.4)	— **<0.001**	1 4.00 (2.27–7.04)
**Number of days of treatment for COVID‐19** (days)							
≤7 8–14 >14	374 (48.9) 371 (48.6) 19 (2.5)	136 (36.4) 143 (38.5) 11 (57.9)	— 0.539 0.066	1 1.10 (0.82–1.48) 2.41 (0.94–6.13)	93 (24.9) 108 (29.1) 9 (47.4)	— 0.192 **0.035**	1 1.24 (0.90—1.72) 2.72 (1.07–6.90)
**Time since recovery to study entry** (weeks)							
≤4 4–8 8–12	106 (13.9) 290 (37.9) 368 (48.2)	44 (41.5) 112 (38.6) 134 (36.4)	0.603 0.340	1 0.89 (0.56–1.39) 0.81 (0.52–1.25)	34 (32.1) 80 (27.6) 96 (26.1)	— 0.383 0.224	1 0.81 (0.50–1.31) 0.75 (0.47–1.19)

*Note:* Values in bold indicate statistically significant results (*p* < 0.05).

^a^ Univariate logistic regression.

Multivariable analysis (Table 1) revealed that receiving ≥3 vaccine doses was significantly protective against both reduced quality of life (OR = 0.31, 95% CI: 0.13–0.77) and social stigma (OR = 0.2, 95% CI: 0.08–0.51). Conversely, moderate‐to‐severe acute symptoms were strong predictors for both outcomes (QoL decline: OR = 2.55; stigma: OR = 4.0). Although comorbidities (OR = 1.65; 95% CI: 1.11–2.45) and hospitalization (OR = 2.25; 95% CI: 1.05–4.82) significantly predicted impaired post‐discharge quality of life, they did not correlate with social stigma. Conversely, treatment duration >14 days was associated with higher perceived stigma (OR = 2.72; 95% CI: 1.07–6.90) but showed no significant link to quality‐of‐life decline (Table 1).

### 3.3 Association Between Persistent Symptoms of COVID‐19 and Perceived Social Stigma and Reduce in Quality of Life

Overall, 81.3% of study participants reported at least one post‐COVID‐19 symptom following recovery (Supporting information Appendix 1). As shown in Table 2, all eight symptom groups were significantly associated with a decline in quality of life. With respect to perceived social stigma, significant associations were observed for seven of the eight symptom groups, general, otolaryngological, respiratory, cardiovascular, gastrointestinal, and musculoskeletal symptoms (all *p* < 0.05). Neurological symptom groups were not significantly associated with perceived social stigma (Table 2).

**TABLE 2:** Prolonged COVID‐19 symptoms among 764 study participants recovering from COVID‐19 infection, stratified by perceived reduce in quality of life and social stigma.

		**Perceived reduced in quality of life**			**Perceived social stigma**		
Symptom groups	Total *N* = 764 (%)	Decline *n* = 290 (38%)	*p* ^a^ value	OR^a^ (95% CI)	Yes *n* = 210 (27.5%)	*p* ^a^ value	OR^a^ (95% CI)
**General symptoms**							
No Yes	369 (48.3) 395 (51.7)	57 (15.4) 233 (59.0)	**<0.001**	1 7.87 (5.57–11.13)	73 (19.8) 137 (34.7)	— **<0.001**	1 2.15 (1.55–3.00)
**Neurological symptoms**							
No Yes	334 (43.7) 430 (56.3)	46 (13.8) 244 (56.7)	**<0.001**	1 8.21 (5.70–11.83)	81 (24.3) 129 (30.0)	— 0.078	1 1.34 (0.97–1.85)
**Otolaryngology symptoms**							
No Yes	541 (70.8) 223 (29.2)	160 (29.6) 130 (58.3)	**<0.001**	1 3.33 (2.41–4.60)	123 (22.7) 87 (39.0)	— **<0.001**	1 2.17 (1.55–3.04)
**Respiratory symptoms**							
No Yes	406 (53.1) 358 (46.9)	94 (23.2) 196 (54.7)	**<0.001**	1 4.02 (2.94–5.48)	88 (21.7) 122 (34.1)	— **<0.001**	1 1.87 (1.35–2.58)
**Cardiovascular symptoms**							
No Yes	652 (85.3) 112 (14.7)	214 (32.8) 76 (67.9)	**<0.001**	1 4.32 (2.81–6.64)	166 (25.5) 44 (39.3)	— **0.003**	1 1.89 (1.25–2.88)
**Digestive symptoms**							
No Yes	626 (81.9) 138 (18.1)	186 (29.7) 104 (75.4)	**<0.001**	1 7.24 (4.74–11.05)	155 (24.8) 55 (39.9)	— **<0.001**	1 2.01 (1.37–2.96)
**Musculoskeletal symptoms**							
No Yes	648 (84.8) 116 (15.2)	204 (31.5) 86 (74.1)	**<0.001**	1 6.24 (3.99–9.76)	169 (26.1) 41 (35.3)	— **0.041**	1 1.55 (1.02–2.36)
**Dermatological symptoms**							
No Yes	726 (95.0) 38 (5.0)	266 (36.6) 24 (63.2)	**0.001**	1 2.96 (1.51–5.83)	192 (26.5) 18 (47.4)	— **0.006**	1 2.50 (1.30–4.83)

*Note:* Values in bold indicate statistically significant results (*p* < 0.05).

^a^ Univariate logistic regression.

### 3.4 Association Between Perceived Social Stigma and Quality of Life

In univariate analysis, perceived social stigma was strongly associated with reduced quality of life (OR = 2.55, 95% CI 1.84–3.53). After adjusting for age, vaccination status, COVID‐19 severity, and relevant post‐COVID symptom groups such as general symptoms, symptoms otolaryngology, respiratory symptoms, cardiovascular symptoms, digestive symptoms, musculoskeletal symptoms, and dermatological symptoms, perceived social stigma remained independently associated with reduced quality of life (adjusted OR = 1.81; 95% CI: 1.20–2.73) (Table 3). In addition, the multivariable logistic regression results, including the adjustment variables, are presented as odds ratios with 95% confidence intervals in Table 4.

**TABLE 3:** The relationship between quality of life and social stigma among 764 study participants recovering from COVID‐19 infection.

		Quality of life				
Factor	Total *N* = 764 *n* (%)	Decline *n* = 290 (38%)	Univariable logistic regression model		Multivariable logistic regression model	
			*p* value	OR (95% CI)	*p* value^b^	aOR^b^ (95% CI)
**Social stigma**						
No Yes	554 (72.5) 210 (27.5)	177 (31.9) 113 (53.8)	— **<0.001**	1 2.55 (1.84–3.53)	**—** **0.004**	1 1.81 (1.20–2.73)

*Note:* Values in bold indicate statistically significant results (*p* < 0.05).

^b^ Multivariable logistic regression model adjusted with factors: age, number of shots of COVID‐19 vaccine given prior to infection, severity of symptoms with COVID‐19, general symptoms, otolaryngology symptoms, respiratory symptoms, cardiovascular symptoms, digestive symptoms, musculoskeletal symptoms, and dermatological symptoms.

**TABLE 4:** Multivariable logistic regression model adjusted with adjustment variables.

Adjustment variables	Perceived decline in quality of life	
	*p* ^b^ value	OR^b^ (95% CI)
**Age (years)**		
18–30	—	1
31–40	0.600	1.14 (0.70–1.84)
41–50	0.680	1.13 (0.62–2.07)
51–60	0.352	1.32 (0.73–2.38)
61–65	**0.001**	3.85 (1.70–8.68)
**Number of shots of COVID‐19 vaccine given before infection**		
1	—	1
2	0.727	0.81 (0.25–2.63)
3	0.410	0.61 (0.19–1.99)
**Severity of symptoms with COVID‐19**		
Mild		1
Moderate or severe	0.382	0.72 (0.34–1.51)
**General symptoms**		
No	—	1
Yes	**<0.001**	3.97 (2.69–5.86)
**Otolaryngology symptoms**		
No		1
Yes	0.206	1.30 (0.86–1.97)
**Respiratory symptoms**		
No		1
Yes	**<0.001**	2.33 (1.58–3.41)
**Cardiovascular symptoms**		
No		1
Yes	**0.009**	2.01 (1.19–3.38)
**Digestive symptoms**		
No		1
Yes	**<0.001**	3.19 (1.94–5.27)
**Musculoskeletal symptoms**		
No		1
Yes	**<0.001**	3.04 (1.79–5.16)
**Dermatological symptoms**		
No		1
Yes	0.159	1.87 (0.78–4.45)

*Note:* Values in bold indicate statistically significant results (*p* < 0.05).

^b^ Multivariable logistic regression model adjusted with factors: age, number of shots of COVID‐19 vaccine given prior to infection, severity of symptoms with COVID‐19, general symptoms, otolaryngology symptoms, respiratory symptoms, cardiovascular symptoms, digestive symptoms, musculoskeletal symptoms, and dermatological symptoms.

## 4 Discussion

This study provides evidence that post‐COVID‐19 symptoms and perceived social stigma remain substantial challenges among individuals recovering from COVID‐19 in Vietnam, even during the post‐acute phase and the transition to the “new normal.” Our findings have revealed that over 80% of participants reported persistent symptoms, 27.5% experienced social stigma, and 38% reported a decline in quality of life. Notably, perceived social stigma emerged as an independent predictor of impaired quality of life, even after adjusting for sociodemographic factors, vaccination status, disease severity, and persistent symptoms (Adjusted OR = 1.81; 95% CI: 1.20–2.73). These findings highlight that the impact of post‐COVID‐19 sequelae extends beyond physiological dysfunction to encompass significant psychosocial burden. Fear of discrimination and social exclusion may discourage individuals from seeking timely medical or psychological support, thereby amplifying the negative effects of persistent symptoms on daily functioning and health‐related quality of life.

### 4.1 Post‐COVID‐19 Symptoms

The high prevalence of post‐COVID‐19 symptoms observed in this study aligns with emerging global evidence that post‐acute sequelae of SARS‐CoV‐2 infection persist well beyond the acute infection phase [19]. Symptoms across multiple organ systems, including general, respiratory, cardiovascular, gastrointestinal, and musculoskeletal domains, were strongly associated with impaired quality of life [20]. These findings reinforce the concept of post‐COVID‐19 condition as a multisystem disorder that extends beyond physical recovery and interferes with daily functioning, productivity, and overall well‐being.

Our results are consistent with a systematic review and meta‐analysis of 24 studies, which reported that approximately 36% of individuals experienced a decline in quality of life following COVID‐19 recovery, with fatigue and respiratory symptoms being particularly prevalent [21]. That analysis also demonstrated substantial objective sequelae, including abnormal lung function in 39% of patients and persistent radiological abnormalities in up to 59%, such as ground‐glass opacities, septal thickening, fibrous bands, and bronchiectasis [21]. Together with our findings, this evidence underscores the substantial and sustained health burden faced by COVID‐19 survivors.

Although the proportion of participants reporting reduced quality of life in our study (38%) was lower than that reported in studies from Northwest Spain (67%) [22] and the Netherlands (72%) [23], these differences are likely explained by variations in study populations. Participants in those studies were generally older and had more severe acute COVID‐19, whereas most of our participants were younger and experienced mild disease managed at home [22, 23]. This highlights the critical role of age and disease severity in shaping post‐COVID‐19 recovery status.

Notably, neurological symptoms were prevalent but were not significantly associated with perceived social stigma. One possible explanation is that differences in how post‐COVID‐19 symptoms are perceived by others may influence stigmatizing responses. More externally observable symptoms may be more readily interpreted as signs of ongoing illness or infectivity and could, therefore, contribute to social avoidance or discrimination. However, symptom visibility and others' interpretations of symptoms were not directly measured in our study, and this explanation remains speculative [24, 25].

### 4.2 Social Stigma as a Determinant of Quality of Life

Stigma has unintentionally created an invisible barrier between patients and society, leading to a range of negative social consequences [26]. During the COVID‐19 pandemic, stigma emerged as one of the most significant social repercussions of infection [11]. Numerous studies worldwide have documented the prevalence of COVID‐19‐related stigma [27], with reports from developed countries indicating that up to 50% of COVID‐19 survivors experienced discrimination [11], whereas a study in India found that nearly all participants reported at least one stigma‐related experience [28]. Furthermore, a community survey in the United Kingdom revealed an extraordinarily high prevalence of stigma among individuals with post‐COVID conditions, with 95.4% reporting overall stigma, 62.7% experiencing enacted stigma, 86.4% experiencing internalized stigma, and 90.8% experiencing anticipated stigma [29]. In contrast, our study found that approximately 27.5% of individuals recovering from COVID‐19 experienced social stigma, a figure notably lower than that reported in previous studies. This discrepancy may be attributed to differences in cultural contexts, pandemic phases, public health messaging, and the measurement tools used to assess stigma. Nevertheless, our findings confirm that stigma remains a persistent and meaningful issue even as societies transition to post‐pandemic conditions.

Crucially, we identified a statistically significant association between perceived social stigma and reduced quality of life. This relationship has been consistently observed in studies conducted in Iran [30], China [31], and Indonesia [10], where higher stigma levels were associated with poorer quality of life, increased depressive symptoms, and adverse mental health outcomes among COVID‐19 survivors. These converging findings reinforce the interpretation that stigma is not merely a social inconvenience but a key psychosocial determinant of recovery.

Stigma may also exacerbate health outcomes through multiple pathways, including social isolation, reduced access to support networks, internalized shame, and heightened psychological distress. In this way, stigma may interact with post‐COVID symptoms to create a self‐reinforcing cycle that hinders full recovery and reintegration into society [32, 33].

### 4.3 Implications for Public Health and Clinical Practice

Our findings demonstrate that viral clearance is an inadequate metric for clinical recovery, as the health and psychosocial sequelae of COVID‐19 persist well beyond the acute phase. The recovery needs of COVID‐19 survivors are therefore substantial and multifaceted [34]. Healthcare professionals and policy‐makers should prioritize the development of post‐COVID‐19 care strategies that enable early identification and timely management of persistent physical and mental health problems.

In parallel, community‐level interventions are needed to address stigma [27]. Educational campaigns should emphasize that persistent symptoms are not markers of infectivity, thereby dispelling fear‐driven discrimination and fostering social reintegration [5]. Clinically, systematic monitoring within the 3‐month post‐recovery window is essential to detect late‐onset complications, facilitate early intervention, and support post‐COVID‐19 recovery.

### 4.4 Strengths and Limitations

Our study has several limitations that should be acknowledged. First, the cross‐sectional design precludes definitive causal inferences regarding the relationship between perceived social stigma and quality of life. Although using multivariable regression models could control key observed confounders, this statistical adjustment cannot overcome the inherent limitation of our cross‐sectional design. Specifically, cross‐sectional data cannot establish temporal relationships or causality between perceived social stigma, persistent symptoms, and quality of life decline. Hence, longitudinal studies are warranted to delineate the causal pathways and long‐term dynamics among these variables. Second, although the C‐19‐PDS underwent pilot testing and forward‐backward translation to ensure content validity, it lacks formal cultural validation for the Vietnamese population, potentially introducing measurement bias. Third, reliance on self‐reported data for health status and stigma may incur recall bias. We mitigated this by limiting recruitment to a 1–3‐month post‐recovery window and using structured instruments. Third, although perceived stigma may not capture all enacted discrimination, its strong correlation with quality‐of‐life outcomes underscores its clinical relevance. Future research should prioritize longitudinal monitoring of vulnerable cohorts to strengthen societal resilience against subsequent health emergencies [35, 36]. Finally, our sample included a relatively small proportion of participants who had been hospitalized compared with those who had experienced COVID‐19 in the community. This may reflect the younger demographic and the predominance of mild infections in the community setting. However, this feature may limit the generalizability of our results to older populations or individuals with more severe acute COVID‐19 requiring hospitalization or intensive care. Therefore, caution is warranted when comparing our findings with cohorts of hospitalized or elderly patients, in whom the prevalence and severity of post‐COVID‐19 symptoms and their impact on quality of life may differ.

## 5 Conclusions

Post‐COVID‐19 symptoms are common among COVID‐19 survivors in Vietnam, and social stigma remains prevalent and significantly associated with reduced quality of life in the post‐recovery period. Addressing both physical sequelae and social stigma is essential to support full recovery and reintegration into society. Public health strategies should prioritize post‐COVID‐19 care, mental health support, stigma reduction, and community education. Lessons learned from the COVID‐19 pandemic highlight the importance of preparing health systems and societies to address not only biomedical but also the social consequences of future public health crises.

## Author Contributions

**Binh‐Minh Le Thi Nguyen**: project administration, conceptualization, methodology, investigation, data curation, formal analysis, visualization, writing – original draft. **Truc Thanh Thai**: supervision, conceptualization, methodology, validation, writing – review and editing. **Tri Nugraha Susilawati**: methodology, validation, visualization, writing – review and editing. **Thanh Chi Vo**: resources, visualization, validation, writing – original draft. **Mai Quynh Pham**: investigation, visualization, writing – original draft. **Anh‐Thu Trang Thai**: investigation, formal analysis, writing – original draft. **Nguyen Thao Dang**: investigation, formal analysis, writing – original draft. **Nhan My Le**: investigation, writing – original draft. **Thuy Anh Bui**: validation, visualization, methodology, writing – review and editing, funding acquisition. **Minh Cuong Duong**: supervision, conceptualization, methodology, formal analysis, writing – review and editing, funding acquisition. All authors approved the final manuscript. The authors accept full accountability for the accuracy and integrity of all aspects of this work, ensuring that any concerns are thoroughly investigated and resolved.

## Funding

The authors have nothing to report.

## Ethics Statement

The study was reviewed and approved by the Ethics Committee of the University of Medicine and Pharmacy at Ho Chi Minh City (IRB# 244/ĐHYD‐HĐĐĐ).

## Consent

Informed consent was obtained from all individual participants included in the study. Prior to completing the survey, participants were fully informed about the study's objectives, data confidentiality, and their right to withdraw at any time without penalty.

## Conflicts of Interest

The authors declare no conflicts of interest.

## Supporting information



Supporting File 01: puh270396‐sup‐0001‐SuppMat.docx

## Data Availability

Data analyzed in this study are available upon reasonable request to the corresponding author. Access requires commitment to ethical and responsible use protocols to ensure the protection of sensitive information.
