# Supplementary material for: Post‐COVID‐19 Public Health Challenges: Insights Into Post‐COVID Symptoms, Stigma, and Post‐Pandemic Recovery Support From the Vietnam Experience
Source: Public Health Chall. 2026 Oct 4;5(4):e70396. doi: 10.1002/puh2.70396 (PMC13634555; doi:10.1002/puh2.70396)
Supplement: Supplementary file 1 [file PUH2-5-e70396-s001.docx]

**POST-COVID-19 PUBLIC HEALTH CHALLENGES: INSIGHTS INTO PROLONGED SYMPTOMS, STIGMA, AND POST-PANDEMIC RECOVERY SUPPORT FROM THE VIETNAM EXPERIENCE**

**Author list**

*Binh-Minh Le Thi Nguyen^1,2^, Truc Thanh Thai^1^, Tri Nugraha Susilawati^4^, Thanh Chi Vo^1^, Mai Quynh Pham^1^,
Anh-Thu Trang Thai^1^, Nguyen Thao Dang^1^, Nhan My Le^1^, Thuy Anh Bui^3^, Minh Cuong Duong^3^*

**Institutional Affiliations**

^1^ University of Medicine and Pharmacy at Ho Chi Minh City, 217 Hong Bang Street, Ward 11, District 5, Ho Chi Minh City, Vietnam.

^2^ Nguyen Trai Hospital, 314 Nguyen Trai, An Dong Ward, Ho Chi Minh City, Vietnam.

^3^ School of Population Health, Building C29 HTH Level 5, UNSW Sydney, NSW, 2052 Australia.

^4^ Faculty of Medicine, Universitas Sebelas Maret, Jl. IR. Sutami 36 Surakarta, Indonesia.

***The corresponding author:*** *Minh Cuong Duong, PhD, School of Population Health, Building C29 HTH Level 5, UNSW Sydney, NSW, 2052 Australia (telephone: +61 (2) 9385 1185, email:* [*minh.duong@unsw.edu.au*](mailto:minh.duong@unsw.edu.au)*), ORCID: 0000-0002-9300-0047*

**Appendix 1**. Percentage of persistent COVID-19 symptoms in the study sample (N=764)

| **Persistent COVID-19 symptoms** | **Frequency (Percent)** |
| --- | --- |
| *General symptoms (**Pain,* *anxiety,* *fatigue,* *hair loss,* *fever)* | |
| Pain | 116 (15,2) |
| Anxiety | 69 (9,0) |
| Fatigue | 239 (31,3) |
| Hair loss | 181 (23,7) |
| Fever | 82 (10,7) |
| *Neurological symptoms (**Needles/numbness, dizziness, headache,* *forgetfulness, loss of concentration,* *sleep disturbance)* | |
| Needles/numbness | 46 (6,0) |
| Dizziness | 92 (12,0) |
| Headache | 175 (22,9) |
| Forgetfulness | 265 (34,7) |
| Loss of concentration | 138 (18,1) |
| Sleep disturbance | 147 (19,2) |
| *Otolaryngology symptoms (**Earache,* *dry throat, loss of smell,* *loss of taste, tinnitus)* | |
| Earache | 4 (0,5) |
| Dry throat | 128 (16,7) |
| Loss of smell | 79 (10,3) |
| Loss of taste | 84 (11,0) |
| Tinnitus | 31 (4,1) |
| *Respiratory symptoms (Cough, shortness of breath)* | |
| Cough | 304 (39,8) |
| Shortness of breath | 120 (15,7) |
| *Cardiovascular symptoms (Chest pain/tightness, palpitations)* | |
| Chest pain/tightness | 69 (9,0) |
| Palpitations | 66 (8,6) |
| *Digestive symptoms (**Nausea,* *Anorexia,* *Abdominal pain,* *Diarrhea)* | |
| Nausea | 35 (4,6) |
| Anorexia | 81 (10,6) |
| Abdominal pain | 27 (3,7) |
| Diarrhea | 49 (6,4) |
| *Musculoskeletal symptoms (**Joint pain, muscle pain)* | |
| Joint pain, muscle pain | 116 (15,2) |
| *Dermatological symptoms (Rash,* *dermatitis)* | |
| Rash | 10 (1,3) |
| Dermatitis | 32 (4,2) |
| **No symptoms** | **143 (18,7)** |
